# Revision of the genus *Parasapyga* Turner (Hymenoptera, Sapygidae), with the description of two new species

**DOI:** 10.3897/zookeys.369.6691

**Published:** 2014-01-13

**Authors:** Cornelis van Achterberg

**Affiliations:** 1 Department of Terrestrial Zoology, Naturalis Biodiversity Center, Postbus 9517, 2300 RA Leiden, The Netherlands

**Keywords:** Revision, Sapygidae, Parasapyga, key, new species, Oriental, Indonesia, Vietnam

## Abstract

Two new species, *Parasapyga boschi* **sp. n.** from Vietnam and *P. yvonnae* **sp. n.** from Indonesia are described. *Parasapyga walshae* van der Vecht, 1940, is treated as a valid species instead of a subspecies of *P. moelleri* Turner, 1910. A key to the species of the genus is added and all species are illustrated.

## Introduction

**Figure 1.**
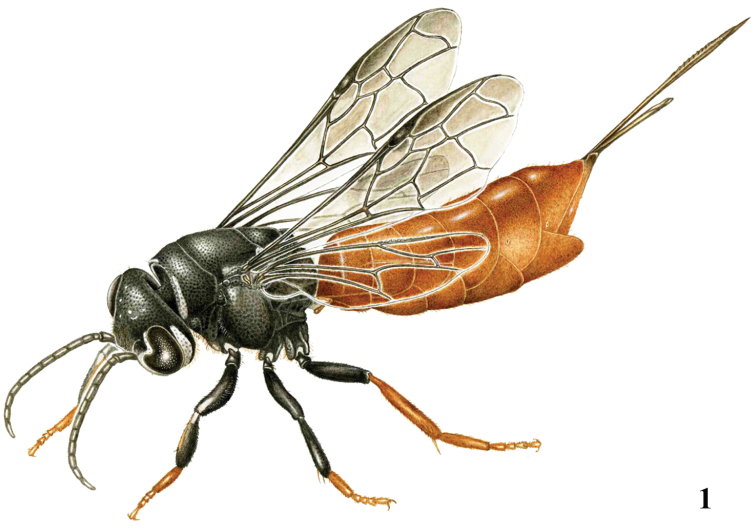
*Parasapyga boschi* sp. n., holotype, ♀, habitus dorso-lateral. Illustration: Erik-Jan Bosch.

The little known aculeate family Sapygidae (Hymenoptera) is rarely collected and wide-spread in the Holarctic Region, but rare in other regions and unknown from the Australian Region. There are approx. 70 described extant species distributed among 12 extant genera (Kurzenko 1995, 1996, Bennett and Engel 2005, Huber 2009) in two subfamilies. Despite belonging to the Aculeata the females possess an ovipositor; in the subfamily Sapyginae with a serrate dorso-apical part (Figs 3, 12, 40) and the sheath without setae subapically (Figs 1, 3), vein 2r-m of the fore wing is distinctly sinuate (Fig. 4) and the eyes deeply incised at the inner side (Fig. 1). In the subfamily Fedtschenkiinae the ovipositor has no serrate part and the sheath has subapical setae, vein 2r-m of the fore wing is weakly curved and the eyes are not incised. Sapyginae occur where its host is nesting, including the home-made bee hotels in gardens; Fedtschenkiinae occur in deserts or salt steppes. According to Brothers (1975) Sapygidae are the sister-group of the Mutillidae *sensu lato.* Recent research indicates that the sister-group of the Sapygidae is the family Myrmosidae and together they are sister to the Mutillidae (Pilgrim et al. 2008). There are only keys to genera for the Palaearctic, Holarctic and Neotropical regions by Kurzenko and Gusenleitner (1994), Kurzenko (1996) and Brothers (2006), respectively.

**Figures 2–3.**
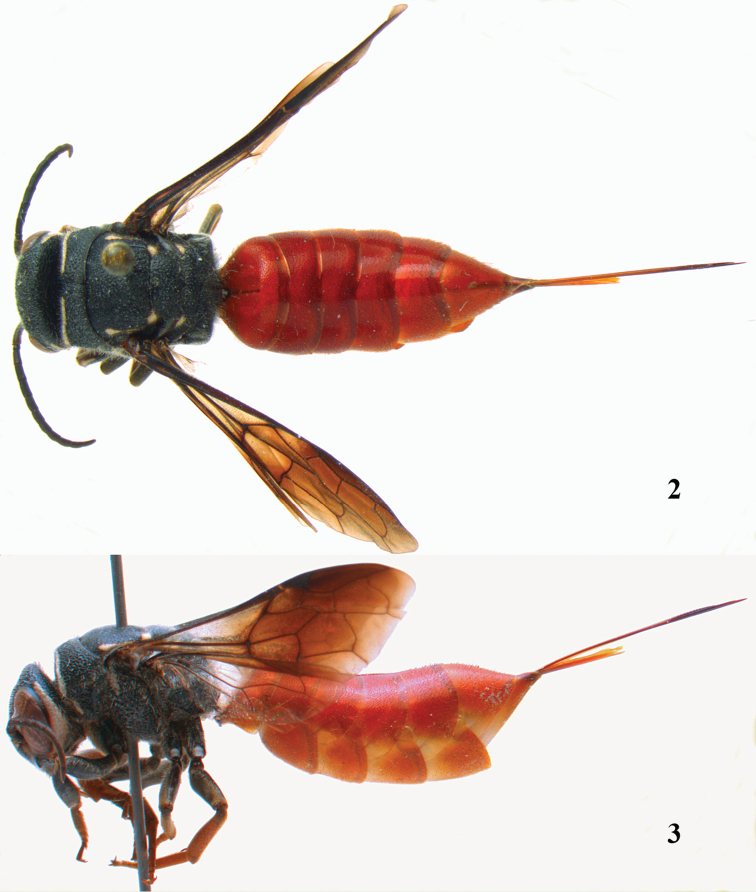
*Parasapyga boschi* sp. n., holotype, female. **2** habitus dorsal **3** habitus lateral.

**Figures 4–12.**
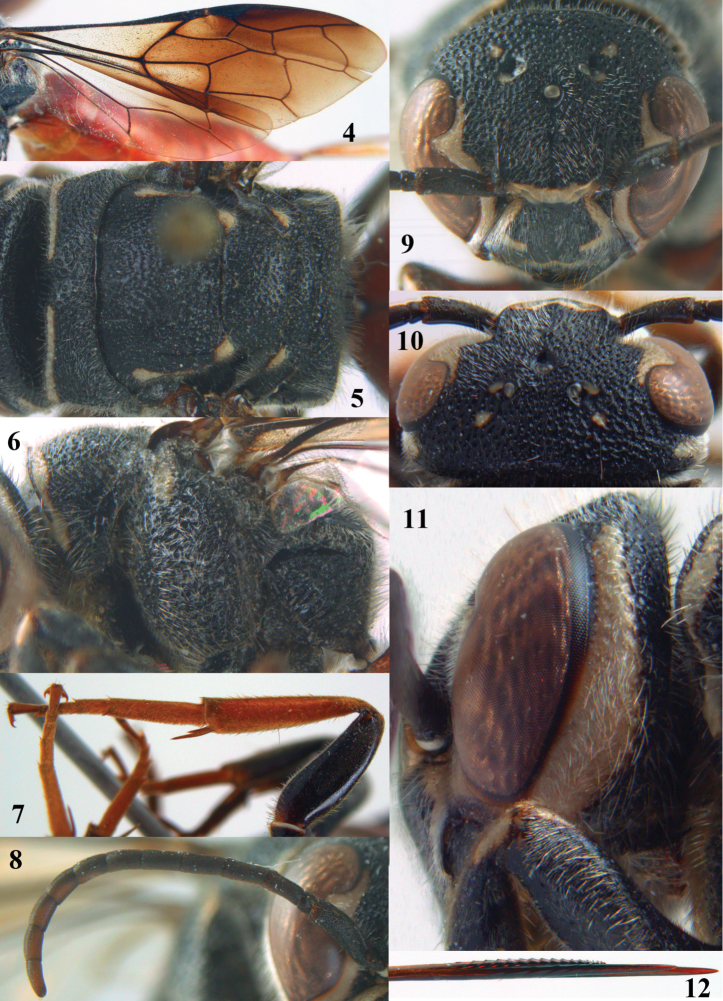
*Parasapyga boschi* sp. n., holotype, female. **4** wings **5** mesosoma dorsal **6** mesosoma lateral **7** hind leg lateral **8** antenna lateral **9** head anterior **10** head dorsal **11** head lateral **12** apex of ovipositor lateral.

In the Oriental Region Sapygidae are very rarely collected and with few species in only three genera present (Kurzenko 1996): *Sapyga* Latreille, 1796 (one species) and *Parasapyga* Turner, 1910, with only the type species *Parasapyga moelleri* Turner, 1910, India (Sikkim) and one additional known subspecies from Indonesia (Sumatra; *Parasapyga moelleri walshae* van der Vecht, 1940), and “*Polochrum*” *flavicolle* Cameron, 1899, from North India (Sikkim). The generic position of the latter is uncertain (Kurzenko in litt.). However, the oldest known fossil of the Sapygidae is known from the Oriental Region; *Cretosapyga resinicola* Bennett & Engel, 2005. It was found in mid-Cretaceous (latest Albian, ca. 100 Mya) amber from Myanmar (Bennett and Engel 2005). The fossil is placed in the new extinct subfamily Cretosapyginae Bennett & Engel, 2005. The genus *Parasapyga* can be recognised from the Holarctic genera of Sapyginae by having the clypeus extending dorsally to the frontal shelf anteriorly (Figs 9, 41), the head without calli or welts and the ocelli medium-sized (Figs 10, 42).

The biology of *Parasapyga* species is unknown, but other Sapyginae are cleptoparasitoids (or predator-inquilines) of solitary bees (belonging to Apinae *sensu lato* and Megachilinae *sensu lato*). The female wasp oviposits into the nest cell of host, the larva consumes first the host egg or larva followed by the food supply of the bee larva (Rozen and Kamel 2009). Members of the small monotypic subfamily Fedtschenkiinae parasitize larvae of ground-nesting Eumeninae (Vespidae) after the larva has spun its cocoon.

## Taxonomy

### Parasapyga

Turner, 1910

http://species-id.net/wiki/Parasapyga

Figs 1 –43

Parasapyga Turner 1910: 405; van der Vecht 1940: 45; Kurzenko 1996: 90. Type species (by monotypy): *Parasapyga moelleri* Turner, 1910.

#### Diagnosis.

Clypeus extending dorsally to the frontal shelf anteriorly, resulting in absence of face medially (Figs 9, 41); inner orbit of eye without callus or welt (Figs 41, 42); ocelli medium-sized (Figs 10, 42); outer side of eye evenly convex medially (Fig. 41-43); occipital carina absent; length of malar space about half apical width of scapus (Fig. 9); entire propodeum densely and rather coarsely reticulate-rugose (Figs 5, 26, 36); third submarginal cell of fore wing anteriorly distinctly narrower than posteriorly and vein 2r-m distinctly sinuate (Figs 4, 25, 35); vein cu-a of fore wing interstitial and inclivous (Figs 4, 25); hind coxa without longitudinal carina dorsally (Fig. 34); hypopygium of female evenly convex ventrally. Males unknown.

**Figures 13–14.**
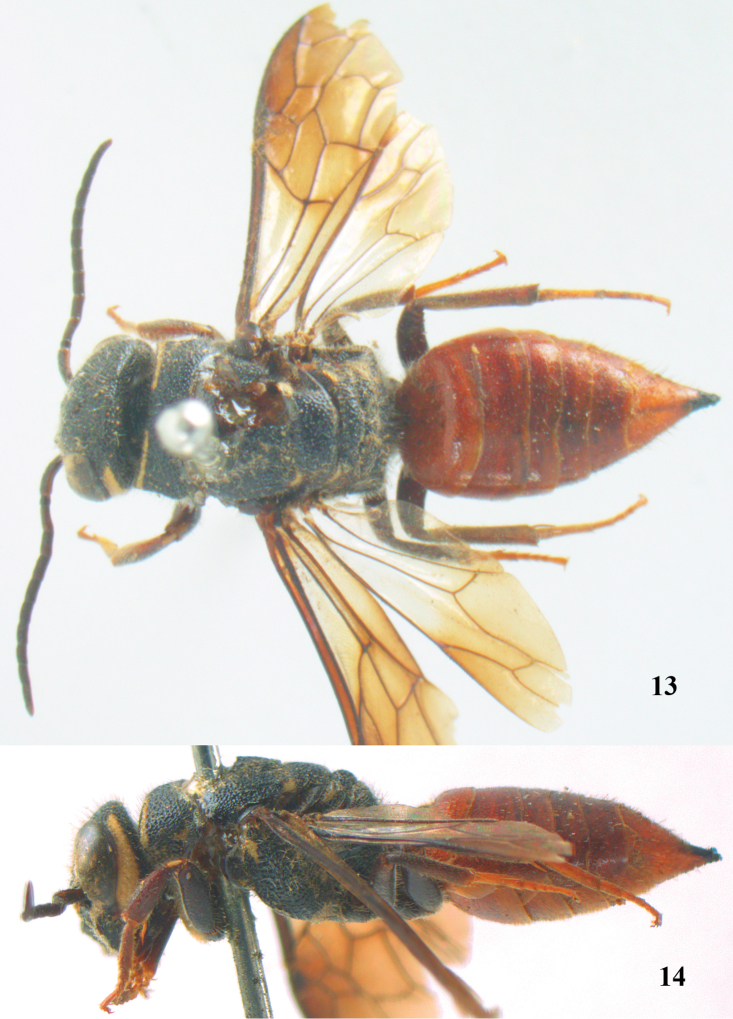
*Parasapyga moelleri* Turner, holotype, female. **13** habitus dorsal **14** habitus lateral.

**Figures 15–22.**
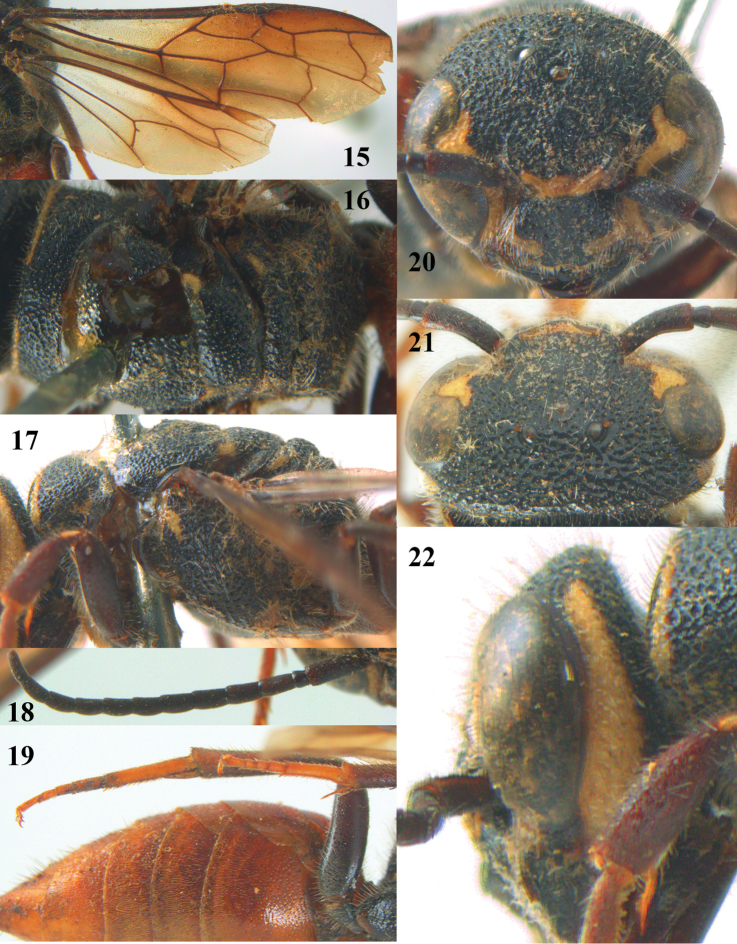
*Parasapyga moelleri* Turner, holotype, female. **15** wings **16** mesosoma dorsal **17** mesosoma lateral **18** antenna lateral **19** hind leg lateral **20** head anterior **21** head dorsal **22** head lateral.

**Figures 23–24.**
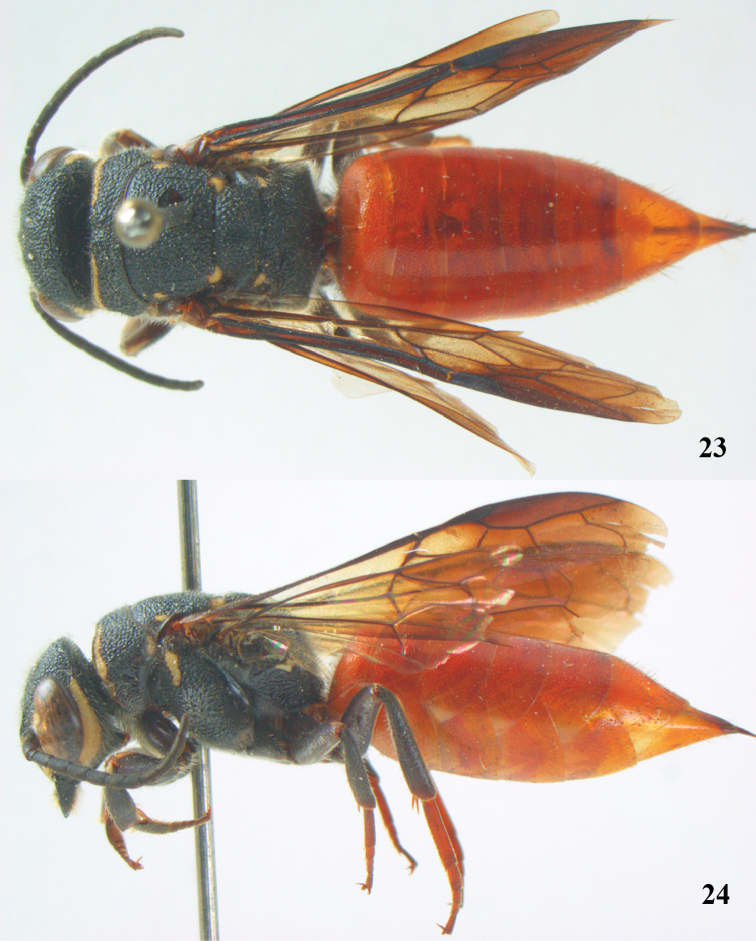
*Parasapyga walshae* van der Vecht, holotype, female. **23** habitus dorsal **24** habitus lateral.

**Figures 25–32.**
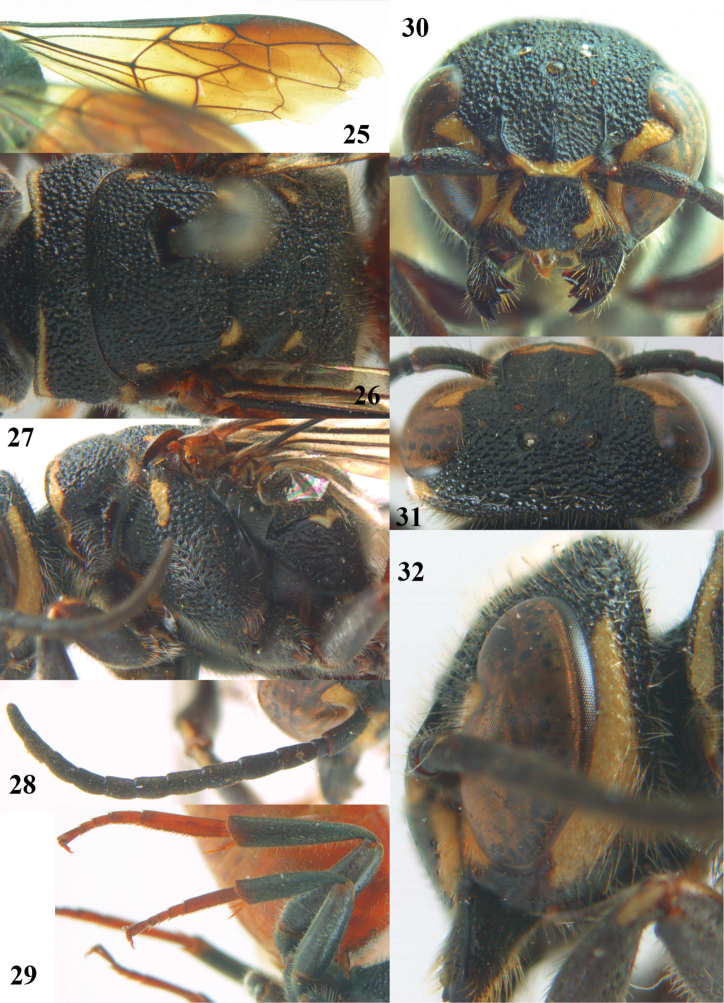
*Parasapyga walshae* van der Vecht, holotype, female. **25** wings **26** mesosoma dorsal **27** mesosoma lateral **28** antenna lateral **29** hind leg lateral **30** head anterior **31** head dorsal **32** head lateral.

**Figures 33–34.**
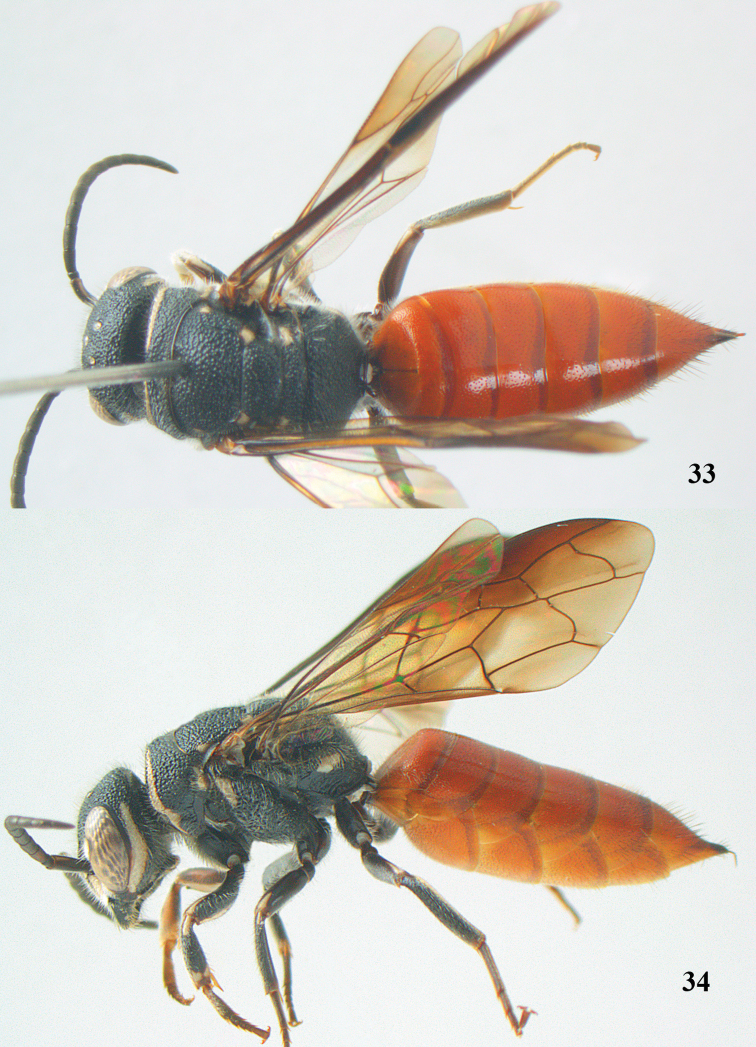
*Parasapyga yvonnae* sp. n., holotype, female. **33** habitus dorsal **34** habitus lateral.

**Figures 35–43.**
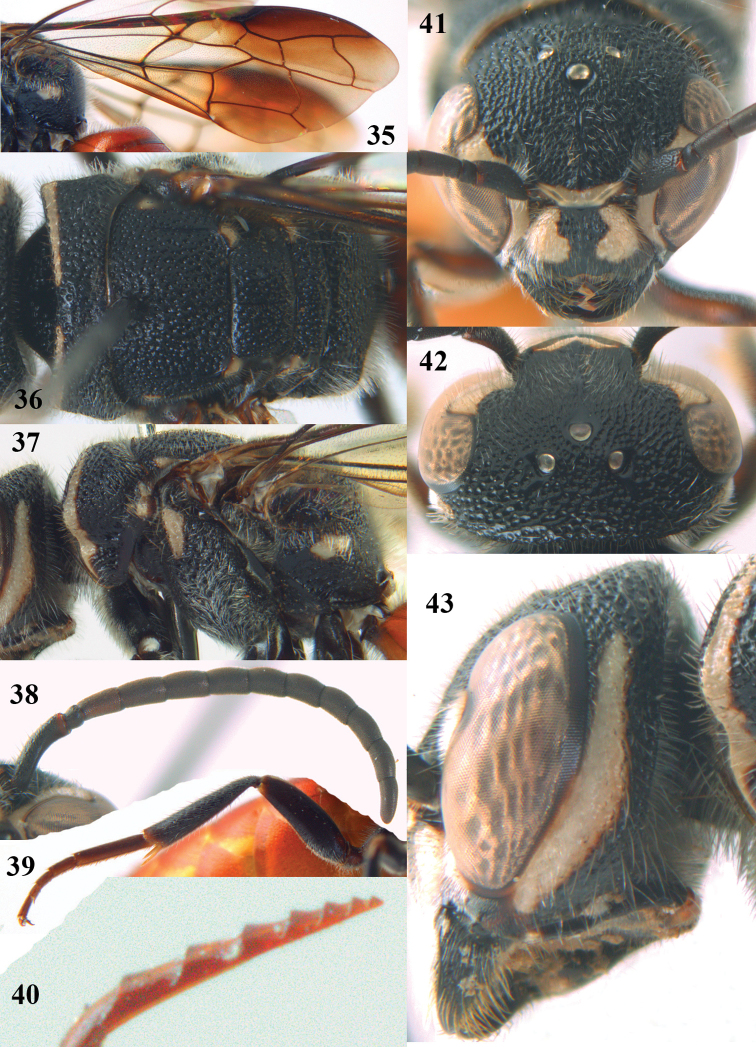
*Parasapyga yvonnae* sp. n., holotype, female. **35** wings **36** mesosoma dorsal **37** mesosoma lateral **38** antenna lateral **39** hind leg lateral **40** apex of ovipositor lateral **41** head anterior **42** head dorsal **43** head lateral.

#### Biology.

Unknown.

#### Distribution.

Oriental (four species).

#### Key to species of the genus *Parasapyga* Turner

**Table:** 

1	Clypeus with narrow anchor-shaped black patch medially (Fig. 41); ovipositor with rather widely separated serrations (Fig. 40); metasoma rather slender in dorsal view (Fig. 33); first discal cell of fore wing subhyaline (Fig. 35); North Sumatra	*Parasapyga yvonnae* sp. n.
–	Clypeus with wide anchor-shaped black patch medially (Figs 9, 20, 30); ovipositor densely serrate dorsally (Fig. 12); metasoma rather wide in dorsal view (Figs 13, 23), but slenderer in *Parasapyga boschi* (Fig. 2); first discal cell of fore wing at least laterally distinctly infuscate (Figs 4, 15, 25)	2
2	Ivory patch at incision of eye extended nearly up to level of posterior ocelli (Fig. 9); pair of ivory spots besides posterior ocellus present (Fig. 10); penultimate antennal segment of female 1.1 times as wide as apical segment in dorsal view (Fig. 8); smooth interspaces between punctures of pronotum and metanotum medio-dorsally about equal to diameter of punctures (Fig. 5); ivory transverse stripe of pronotum narrowly interrupted medio-dorsally (Fig. 5); five apical segments of antenna partly brown ventrally (Fig. 8); first subdiscal of fore wing laterally darker than medially (Fig. 4); hind tibia reddish-brown (Fig. 7); South Vietnam	*Parasapyga boschi* sp. n.
–	Ivory patch at incision of eye at most extended up to level of anterior ocellus (Figs 20, 30); pair of ivory spots besides posterior ocellus absent (Fig. 31), at most with a minute patch (Fig. 21); penultimate antennal segment of female 1.2 times as wide as apical segment in dorsal view (Fig. 28); smooth interspaces between punctures of pronotum and metanotum medio-dorsally distinctly narrower than diameter of punctures (Figs 16, 26); ivory transverse stripe of pronotum widely interrupted medio-dorsally (Figs 16, 26); at most one apical segment of antenna brown ventrally and other segments black (Fig. 18); first subdiscal of fore wing laterally as dark as medially (Figs 15, 25); hind tibia dark brown or black (Figs 19, 29)	3
3	Ivory patch at incision of eye remains far from level of anterior ocellus (Fig. 30); dorsally pronotum without smooth and shiny interspaces (Fig. 26); propodeum with irregular ivory patch latero-dorsally (Fig. 27); hind basitarsus rather robust (Fig. 29); clypeus ivory latero-dorsally (Fig. 30); first discal cell of fore wing subhyaline (Figs 23, 25); South Sumatra	*Parasapyga walshae* van der Vecht, 1940
–	Ivory patch at incision of eye at most extended nearly up to level of anterior ocellus (Fig. 20); dorsally pronotum with smooth and shiny convex interspaces (Fig. 16); propodeum entirely black latero-dorsally (Fig. 17); hind basitarsus less robust (Fig. 19); clypeus largely black latero-dorsally (Fig. 20); first discal cell of fore wing dark brown (Figs 13, 15); North India (Sikkim)	*Parasapyga moelleri* Turner, 1910

### Parasapyga boschi sp. n.

http://zoobank.org/28DE635A-46B2-4E7E-896D-8FBC576FB0F1

http://species-id.net/wiki/Parasapyga_boschi

Figs 1 –12

#### Type material.

Holotype, ♀ (RMNH), “S. Vietnam: Dông Nai, Cát Tien N. P., c. 100 m, 19–25.iv.2007, Mal. traps, Dong trail, Mai Phu Quy & Nguyen Thanh Manh, RMNH’07”.

#### Diagnosis.

Clypeus with wide anchor-shaped black patch medially (Fig. 9); ivory patch at incision of eye extended nearly up to level of posterior ocelli (Fig. 9); pair of ivory spots besides posterior ocellus present (Fig. 10); smooth interspaces between punctures of pronotum and metanotum medio-dorsally about equal to diameter of punctures (Fig. 5); ivory transverse stripe on pronotum narrowly interrupted medio-dorsally and comparatively wide ventrally (Figs 5, 6); first discal cell of fore wing distinctly infuscate (Fig. 4); first subdiscal of fore wing laterally darker than medially (Fig. 4); hind tibia reddish-brown (Fig. 7); ovipositor densely serrate dorsally (Fig. 12). Resembles most *Parasapyga walshae* and *Parasapyga moelleri*; it can be easily separated by the larger ivory patch at the incision of the eye (Fig. 9 vs Figs 20, 30) and the reddish-brown hind tibia (Fig. 7 vs Figs 19, 29).

#### Description.

Holotype, ♀, length of body 18.7 mm (of fore wing 11.8 mm).

*Head*. Antenna with 12 segments and penultimate segment 1.1 times as wide as apical segment in dorsal view (Fig. 8); frons coarsely reticulate; vertex coarsely punctate and with distinct smooth interspaces (Fig. 10); temple coarsely punctate and with wide smooth interspaces; malar space densely punctulate; head narrowed behind eyes (Fig. 10); clypeus spaced punctate and with complete median crest (Fig. 9).

*Mesosoma*. Length of mesosoma 1.5 times its height (Fig. 3); mesopleuron largely coarsely reticulate-punctate with narrow smooth interspaces; metapleuron densely punctulate anteriorly and coarsely obliquely rugose posteriorly, with a narrow smooth shiny band above it (Fig. 6); pronotum, mesoscutum, scutellum and metanotum coarsely punctate, medially interspaces between punctures about as wide as punctures and sparsely punctulate (Fig. 5); metanotum medially moderately convex and not protruding above level of scutellum (Fig. 3); entire propodeum densely and coarsely reticulate-punctate, medially hardly coarser than laterally (Fig. 5).

*Wings*. Fore wing: vein 2m-cu moderately postfurcal (Fig. 4).

*Legs*. Hind basitarsus rather robust (Fig. 7).

*Metasoma*. Metasoma rather slender in dorsal view (Fig. 2); basal tergites finely punctate and shiny, with smooth interspaces wider than diameter of punctures (Fig. 2); hypopygium 1.3 times as long as fifth sternite ventrally (Fig. 3); ovipositor densely serrate (Fig. 12); ovipositor sheath and ovipositor far exserted (Figs 1, 3).

*Colour*. Black; ivory: pair of L-shaped lateral patches on clypeus (resulting in a wide black anchor medially), patch at inner orbita of eye extended nearly up to level of posterior ocelli (Fig. 9), shelf of frons anteriorly between antennal sockets, temple largely except narrowly dorsally (Fig. 11), pair of small spots besides posterior ocellus, transverse stripe on pronotum (but narrowly interrupted medially; Fig. 5) and ventrally widened (Fig. 6), small patch on pronotum postero-dorsally, elongate patch on mesopleuron antero-dorsally, elongate patch near tegula, axilla, lateral patch of metanotum and elongate apical patch on fore femur; metasoma orange red; inner side of fore femur and tibia, tarsi and hind tibia largely reddish-brown; fore coxa densely yellowish setose ventrally; veins and pterostigma, dark brown; basal cells, middle of first subdiscal cell and basal half of first submarginal cell of fore wing subhyaline or slightly infuscate; remainder of fore wing dark brown (Fig. 4).

*Male*. Unknown.

#### Distribution.

Vietnam.

#### Etymology.

Named in honour of the scientific illustrator, Erik-Jan Bosch (Leiden) because of his excellent illustrations of Hymenoptera.

### Parasapyga moelleri

Turner, 1910

http://species-id.net/wiki/Parasapyga_moelleri

Figs 13 –22

Parasapyga mölleri Turner 1910: 405–406, Pl. L-8; van der Vecht 1940: 45.

#### Type material.

Holotype, ♀ (BMNH), “Type”, “[India], Sikkim, Tukvar, 4000 ‘[ft], iv.[19]01, ex Möller, Bingham Coll.”, “*Parasapyga mölleri* Turn., Type”, “B.M. Type Hym. 15.1268”.

#### Diagnosis.

Clypeus with wide anchor-shaped black patch medially and largely black latero-dorsally (Fig. 20); ivory patch at incision of eye nearly extending up to level of anterior ocellus (Fig. 20); pair of ivory spots besides posterior ocellus absent (Fig. 21); ivory transverse stripe on pronotum widely interrupted medio-dorsally and narrow ventrally (Figs 16, 17); propodeum entirely black latero-dorsally (Fig. 17); first discal cell of fore wing distinctly infuscate and first subdiscal laterally as dark as medially (Figs 13, 15); metasoma rather wide in dorsal view (Fig. 13).

#### Description.

Holotype, ♀, length of body 15.2 mm (of fore wing 10.6 mm).

*Head*. Antenna with 12 segments and penultimate segment 1.2 times as wide as apical segment in dorsal view (Fig. 18); frons rather coarsely reticulate-rugose; vertex coarsely reticulate-punctate (Fig. 21); temple coarsely punctate; malar space densely punctulate; head directly narrowed behind eyes (Fig. 21); clypeus coarsely punctate and dorsally with median crest (Fig. 20).

*Mesosoma*. Length of mesosoma 1.5 times its height (Fig. 14); mesopleuron largely coarsely reticulate; metapleuron densely punctulate anteriorly and coarsely obliquely costate posteriorly, with a moderately wide smooth shiny band above it (Fig. 17); pronotum, mesoscutum, scutellum and metanotum coarsely reticulate-punctate, smooth interspaces between punctures of pronotum and metanotum medio-dorsally mostly distinctly narrower than diameter of punctures (Fig. 16); metanotum medially distinctly convex and distinctly protruding above level of scutellum (Figs 14, 17); entire propodeum densely and rather coarsely reticulate-rugose (Fig. 16).

*Wings*. Fore wing: vein 2m-cu far postfurcal (Fig. 15).

*Legs*. Hind basitarsus rather slender (Fig. 19).

*Metasoma*. Metasoma rather wide in dorsal view (Fig. 13); basal tergites finely punctate and shiny, with smooth interspaces wider than diameter of punctures (Fig. 13); hypopygium 1.2 times longer than fifth sternite ventrally (Fig. 14); ovipositor unknown (broken in holotype).

*Colour*. Black; ivory: L-shaped lateral patch on clypeus, patch at incision of eye extending nearly up to level of anterior ocellus (Fig. 20), shelf of frons anteriorly between antennal sockets, temple (except posteriorly) and up to upper level of eye (Fig. 22), transverse stripe on pronotum (except wide interruption medially; Fig. 16, and narrowed ventrally), small patch on mesopleuron antero-dorsally, minute patch near tegula, axilla, lateral patch on metanotum, apical patch on fore femur and small basal patch on fore tibia; metasoma dark red; palpi brown; tarsi yellowish-brown; remainder of femora and tibiae, veins and pterostigma, dark brown; fore coxa densely golden setose; apical 0.6 of fore wing dark brown and remainder subhyaline (Fig. 15).

*Male*. Unknown.

#### Distribution.

India (Sikkim).

### Parasapyga walshae

van der Vecht, 1940 stat. n.

Figs 23 –32

Parasapyga mölleri walshae van der Vecht, 1940: 45–46, fig.

#### Type material.

Holotype, ♀ (RMNH), “[Indonesia:] S. Sumatra, Res. Lampongs, Mt. Tanggamoes, 22.vii–5.viii.1935, M.E. Walsh”, “*Parasapyga mölleri* Turn. subsp. *walshae* v. d. Vecht, ♀”, “Holotype of subsp. n. *walshae*”.

#### Diagnosis.

Clypeus with wide anchor-shaped black patch medially and ivory latero-dorsally (Fig. 30); ivory patch at incision of eye remains far from level of anterior ocellus (Fig. 30); pair of ivory spots besides posterior ocellus absent (Fig. 31); ivory transverse stripe on pronotum widely interrupted medio-dorsally (Fig. 26) and narrow ventrally (Fig. 27); propodeum with irregular ivory patch latero-dorsally (Fig. 27); first discal cell of fore wing subhyaline and first subdiscal laterally as pale as medially (Figs 23, 25); metasoma rather wide in dorsal view (Fig. 23); ovipositor densely serrate dorsally.

#### Description.

Holotype, ♀, length of body 18.7 mm (of fore wing 11.9 mm).

*Head*. Antenna with 12 segments and penultimate segment 1.2 times as wide as apical segment in dorsal view (Fig. 28); frons moderately reticulate; vertex coarsely reticulate-punctate (Fig. 31); temple coarsely punctate; malar space densely punctulate; head directly narrowed behind eyes (Fig. 31); clypeus coarsely punctate and with complete median crest (Fig. 30).

*Mesosoma*. Length of mesosoma 1.5 times its height (Fig. 24); mesopleuron largely coarsely reticulate; metapleuron densely punctulate anteriorly and coarsely obliquely costate posteriorly, with a narrow smooth shiny band above it (Fig. 27); pronotum, mesoscutum, scutellum and metanotum coarsely punctate-reticulate, interspaces between punctures of pronotum and metanotum medio-dorsally mostly absent (Fig. 26); metanotum medially slightly convex and not protruding above level of scutellum (Fig. 24); entire propodeum densely and rather coarsely reticulate-rugose (Fig. 26).

*Wings*. Fore wing: vein 2m-cu just postfurcal (Fig. 25).

*Legs*. Hind basitarsus rather robust (Fig. 29).

*Metasoma*. Metasoma comparatively wide in dorsal view (Fig. 23); basal tergites finely punctate and shiny, with smooth interspaces wider than diameter of punctures (Fig. 23); hypopygium 1.4 times longer than fifth sternite ventrally (Fig. 24); ovipositor densely serrate dorsally.

*Colour*. Black; ivory: pair of L-shaped lateral patches on clypeus, patch at inner orbita up to top of incision of eye (Fig. 30), shelf of frons anteriorly between antennal sockets, temple except dorsally (Fig. 32), transverse stripe on pronotum (except wide interruption medially; Fig. 26, and narrowed ventrally), small patch on pronotum postero-dorsally, elongate patch on mesopleuron antero-dorsally, patch on border of propodeum and metapleuron, small patch near tegula, axilla, lateral patch on metanotum, elongate apical patch on fore and middle femora and elongate basal patch of fore tibia; metasoma orange red; palpi brown; middle and hind tarsi yellowish-brown; remainder of femora and tibiae, veins and pterostigma, dark brown; fore coxa densely golden setose; first submarginal cell apically, marginal cell and second and third submarginal cells of fore wing dark brown, area below it brown and remainder largely subhyaline (Fig. 25).

*Male*. Unknown.

#### Distribution.

Indonesia (Sumatra).

### Parasapyga yvonnae sp. n.

http://zoobank.org/A9B25C5D-7796-44C5-B14C-8514FFADEEA6

http://species-id.net/wiki/Parasapyga_yvonnae

Figs 33 –43

#### Type material.

Holotype, ♀ (RMNH), “Indonesia: N. Sumatra, Ketambe, c 400 m, near N. P. Gn. Leuser, Mal. trap, vi.1994, Y. v. Nierop & C. v. Achterberg, RMNH’95”.

#### Diagnosis.

Clypeus with rather narrow anchor-shaped black patch medially (Fig. 41); (Fig. 36) and comparatively wide ventrally (Fig. 37); first discal cell of fore wing subhyaline (Fig. 35); hind tibia black; metasoma comparatively slender in dorsal view (Fig. 33); ovipositor with comparatively widely separated serrations (Fig. 40). Differs from the other known species by the rather narrow anchor-shaped black patch of the clypeus, the narrowly interrupted ivory transverse stripe on the pronotum and the dark brown hind tarsus.

#### Description.

Holotype, ♀, length of body 13.9 mm (of fore wing 9.8 mm).

*Head*. Antenna with 12 segments and penultimate segment 1.2 times as wide as apical segment in dorsal view (Fig. 38); frons moderately reticulate; vertex coarsely reticulate-punctate (Fig. 42); temple coarsely punctate; malar space densely punctulate; head narrowed behind eyes (Fig. 42); clypeus rather coarsely reticulate and with nearly complete median crest (Fig. 41).

*Mesosoma*. Length of mesosoma 1.6 times its height (Fig. 34); mesopleuron largely coarsely reticulate-punctate; metapleuron densely punctulate anteriorly and coarsely obliquely rugose posteriorly, with a wide smooth shiny band above it (Fig. 37); pronotum, mesoscutum, scutellum and metanotum coarsely reticulate-punctate, interspaces between punctures of pronotum and metanotum medio-dorsally present, sparsely punctulate and usually 0.5–1.0 times as wide as punctures (Fig. 36); metanotum medially moderately convex and not protruding above level of scutellum (Fig. 34); entire propodeum densely and rather coarsely reticulate-rugose, medially coarser than laterally (Fig. 36).

*Wings*. Fore wing: vein 2m-cu just postfurcal (Fig. 35).

*Legs*. Hind basitarsus comparatively slender (Fig. 39).

*Metasoma*. Metasoma comparatively slender in dorsal view (Fig. 33); basal tergites finely punctate and shiny, with smooth interspaces wider than diameter of punctures (Fig. 33); hypopygium as long as fifth sternite ventrally (Fig. 34); ovipositor with rather widely separated serrations (Fig. 40).

*Colour*. Black; ivory: pair of wide c-shaped lateral patches on clypeus (resulting in a comparatively narrow black anchor medially; Fig. 41), patch at inner orbita up to top of incision of eye (Fig. 41), shelf of frons anteriorly between antennal sockets, temple largely except narrowly dorsally (Fig. 43), transverse stripe on pronotum (but comparatively narrowly interrupted medially (Fig. 36) and ventrally comparatively wide (Fig. 37)), small patch on pronotum postero-dorsally, elongate patch on mesopleuron antero-dorsally, patch on border of propodeum and metapleuron, small patch near tegula, axilla, lateral patch on metanotum, elongate apical patch on femora, fore femur largely ventrally and elongate basal patch on fore and middle tibiae and outer side of fore tibia subapically largely; metasoma orange red; inner side of fore femur and tibia brown; tarsi largely, veins and pterostigma, dark brown; fore coxa densely silvery setose; first submarginal cell apically, marginal cell and second and third submarginal cells of fore wing dark brown, area below it brown and remainder largely subhyaline (Fig. 35).

*Male*. Unknown.

#### Distribution.

Indonesia (Sumatra).

#### Etymology.

Named in honour of one of the collectors, Yvonne van Nierop (Leiden) for all her collecting efforts in N. Sumatra.

## Supplementary Material

XML Treatment for Parasapyga

XML Treatment for Parasapyga boschi

XML Treatment for Parasapyga moelleri

XML Treatment for Parasapyga walshae

XML Treatment for Parasapyga yvonnae
